# Socioeconomic Status and Vascular Access Patency in Hemodialysis: Analysis of Korean National Health Insurance Service Data from 2008 to 2019

**DOI:** 10.3390/jcm14093074

**Published:** 2025-04-29

**Authors:** Jeong-Ik Park, Daehwan Kim, Hyangkyoung Kim, Seung Boo Yang, Sang Jun Park, Young-joo Kwon

**Affiliations:** 1 Department of Surgery, Ulsan University Hospital, University of Ulsan College of Medicine, Ulsan 44033, Republic of Korea; jipark@uuh.ulsan.kr (J.-I.P.); sjpark@uuh.ulsan.kr (S.J.P.); 2 The Better-Way Clinic, Seoul 04715, Republic of Korea; hwaan3@gmail.com; 3 Department of Surgery, Ewha Womans University Medical Center, Ewha Womans University College of Medicine, Seoul 07985, Republic of Korea; 4 Department of Radiology, Nowon Eulji University Hospital, Seoul 01830, Republic of Korea; ysbysb@eulji.ac.kr; 5 Division of Nephrology, Department of Internal Medicine, Korea University College of Medicine, Seoul 08308, Republic of Korea; yjkwon@korea.ac.kr

**Keywords:** socioeconomic status, vascular access, arteriovenous fistula, arteriovenous graft, end-stage kidney disease, patency

## Abstract

**Background**: Socioeconomic status (SES) disparities impact health outcomes, but their effect on vascular access (VA) in hemodialysis patients in Korea remains underexplored. **Methods**: This study evaluated the association between SES and VA outcomes using National Health Insurance Service data from 2008 to 2019. Incident hemodialysis patients were categorized by insurance status into the health insurance group (HG) and medical aid group (MG). The primary endpoint was VA patency, and the secondary endpoint was all-cause mortality, adjusted for demographics, comorbidities, and lifestyle factors. **Results**: Among 86,036 patients, the MG (12.1%) was younger at VA creation (60.4 ± 13.5 vs. 63.1 ± 13.6 years, *p* < 0.001) and had higher rates of comorbidities (all *p* < 0.05 except cancer). Mortality rates per 100 person-years were higher in the MG (11.66 vs. 9.24 for AVF; 17.94 vs. 16.92 for AVG), as was the total procedure frequency (2.10 vs. 1.87, *p* < 0.001), despite similar percutaneous angioplasty counts (1.20 vs. 1.24, *p* = 0.314). **Conclusions**: Lower SES patients exhibited poorer VA patency and higher mortality rates despite equitable healthcare access and cost coverage in Korea. These findings suggest that non-medical factors, such as adherence to treatment and timely intervention, play a critical role in mitigating these disparities.

## 1. Introduction

End-stage kidney disease (ESKD) is a significant public health issue in South Korea. In 2019, 18,642 new patients began renal replacement therapy, with 83.6% choosing hemodialysis. The prevalence of ESKD has doubled since 2010, positioning Korea sixth globally with an incidence of 1816 per million populations [1,2]. Among hemodialysis patients, 78% use autogenous arteriovenous fistulas (AVF), 15% use grafts (AVG), and 5% rely on tunneled catheters. Given the high hemodialysis dependency, addressing vascular access (VA) patency is crucial for improving patient outcomes [3].

Socioeconomic status (SES) serves as a crucial determinant of health outcomes, exerting profound effects across various medical domains. Understanding the interplay between SES and VA outcomes is imperative given the pivotal role of VA in the management of patients requiring hemodialysis, which requires frequent revision, impacting both lifespan and quality of life, and imposing significant social costs [4].

In South Korea, efforts have been made to enhance healthcare accessibility by operating universal health coverage systems composed of a two-tiered system [5]. The lowest-income population is covered by the Medical Aid program, while the remaining population is covered by the National Health Insurance program [6]. Dialysis patients are assigned specific codes under this system [7]. Typically, patients receiving dialysis under healthcare insurance are responsible for 10% of all medical expenses, including invasive procedures, with NHIS covering the remaining 90%. However, some low-income dialysis patients receiving medical aid benefits are also exempt from the 10% co-payment requirement for medical expenses that they would otherwise be responsible for. However, individuals undergoing hemodialysis often face disruptions in their social lives, increasing their vulnerability to lower SES. Consequently, medical aid allocation for ESKD patients is relatively high compared to other health conditions [8].

Despite advancements in renal care, significant knowledge gaps persist regarding the association between SES and VA outcomes, stemming from conflicting results from previous studies, which hinder efforts to address health disparities effectively [8,9,10,11]. This study aimed to assess the association between SES and VA outcomes in a national cohort of hemodialysis patients, aiming to provide comprehensive insights into this critical issue.

## 2. Materials and Methods

### 2.1. Data Sources

This study utilized data obtained from the National Health Insurance Service (NHIS) covering the period from January 2008 to December 2019 [12]. A comprehensive dataset, including demographic information, International Classification of Disease, Tenth Revision (ICD-10) diagnosis codes, procedure codes, prescription details, survival data from both inpatient and outpatient services, and health screening data (including information on smoking or alcohol habits), was meticulously collected and analyzed. Approval for data collection and publication was obtained from the local institutional review board (IRB No. 2020-0576) of Asan medical center, which waived the requirement for written informed consent due to the retrospective nature of the study. All methods adhered to relevant guidelines and regulations.

### 2.2. Study Population and Study Outcome

**Figure 1:**
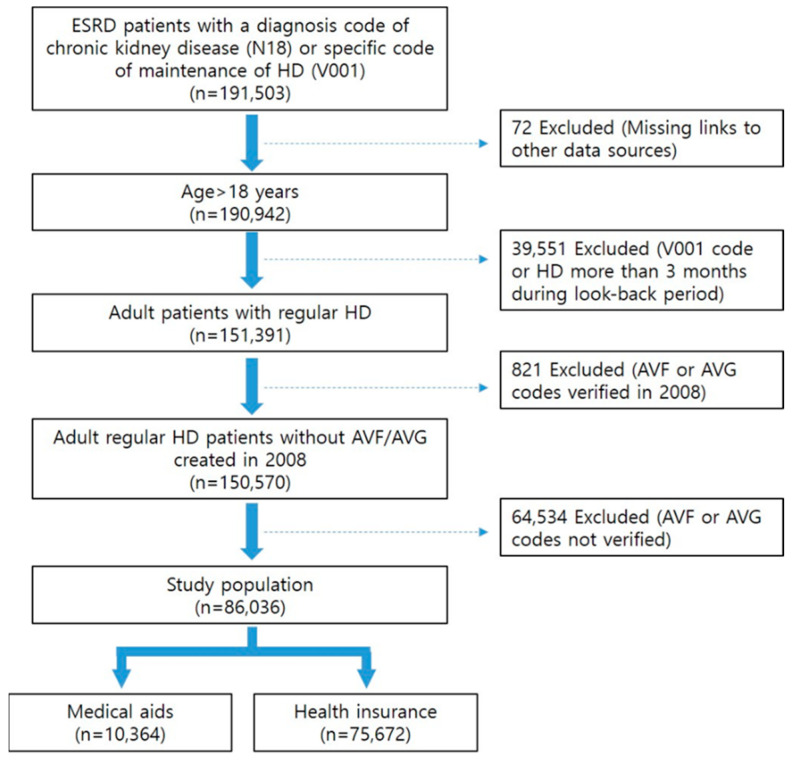
Flow diagram of population selection.

Figure 1 illustrates the flow diagram of the study. Initially, a list of ESKD patients undergoing regular hemodialysis with a diagnosis of advanced chronic kidney disease (N18) or a specific code indicating maintenance of hemodialysis (V001) between 2009 and 2018 was compiled. From this group, incident hemodialysis patients with VA, including arteriovenous fistula (AVF) (procedure code: O2011, O2012, O2081) or arteriovenous graft (AVG) (procedure code: O2082) placed as their initial VA, maintaining hemodialysis for at least three months from the index date (coded as N18 and O7020, O7021, or V001), were identified.

Exclusion criteria were applied to remove patients younger than 18, those with VA creation in 2008, those acquiring the V001 code or undergoing hemodialysis for more than three months during the look-back period, or those lacking relevant VA creation codes.

Patients were categorized into two groups based on their health insurance. The first is the health insurance group (HG), covered by the NHIS, typically bearing 70% of medical expenses, with patients responsible for the remaining 30%. However, the health insurance calculation special exception applies, reducing the patient’s share of expenses to 10% for hemodialysis of chronic kidney disease patients. The medical aid group (MG) consists of recipients of medical aid, catering to individuals unable to afford NHIS premiums.

The index date for each group was defined as the first creation date of VA. Duplicated creations were eliminated, considering them as AVG when both AVF and AVG codes were found on the same date.

The primary endpoint was VA patency, including primary, assisted primary, and secondary patency of AVFs and AVGs, and the incidence rate for 100 person-years. AV access patency was defined following the standards suggested by the Society for Vascular Surgery and operationalized using procedural codes specific to the Korean National Health Insurance system [13]. Primary patency was defined as the interval from AVF or AVG creation to the first intervention, including percutaneous transluminal angioplasty (PTA; procedure code: M6597) or percutaneous thrombectomy (procedure codes: M6632, M6633, M6639), typically accompanied by venography (HA731, HA711, HA712). Assisted primary patency was defined as the interval from access creation to access thrombosis or surgical or percutaneous revision, including surgical thrombectomy/revision (O2083) and percutaneous stent deployment (M6605, M6613). Secondary patency was defined as the interval from access creation to access abandonment, determined by either the creation of a new vascular access or the insertion of a tunneled catheter (O7011, O7012, O7013, O7014) without subsequent removal within one year.

Procedure codes used to define the primary endpoint included percutaneous transluminal angioplasty (PTA; procedure code: M6597), percutaneous thrombectomy (procedure code: M6632, M6633, M6639), surgical thrombectomy or revision (procedure code: O2083), or percutaneous stent deployment (procedure code: M6605, M6613). Claims data unrelated to procedures for VA of hemodialysis, such as angioplasty for renal arteries or stent placement for iliofemoral arteries, by the combined use of fistulography procedure codes (HA731, HA711, HA712, HA732, HA643) were excluded.

The secondary endpoint was patient death, with information on death and death date retrieved from the registration records of the NHIS. The censoring date was determined as the earliest of the following: date of death, date of study outcomes, date of peritoneal dialysis (procedure code: O7061, O7062, O7071, O7072), date of kidney transplantation (ICD-10: Z940), or the end date of the study period (31 December 2019), whichever occurred first.

### 2.3. Covariates/Clinical Variables

Age categories were delineated into four groups: the young age group (18–64 years), the young-old group (65–74 years), the middle-old group (75–84 years), and the oldest-old group (over 85 years). Baseline comorbidities for patients were established using ICD-10 codes within one year of entry, while lifestyle risk factors such as smoking and alcohol consumption were derived from the health screening database. Detailed definitions of covariates can be found in Appendix A Table A1.

### 2.4. Statistical Analysis

Statistical analysis involved presenting demographic data as frequencies for categorical variables and as means with standard deviations for continuous variables. Categorical variables underwent assessment using the Pearson chi-square test, while continuous variables were analyzed using Student’s *t*-test or ANOVA, as appropriate.

The primary, primary assisted, and secondary patency of vascular access, along with patients’ survival, were evaluated using a Kaplan–Meier curve analysis, with group comparisons made using the log-rank test. The Cox proportional hazard regression model was employed to determine adjusted hazard ratios (HR) and corresponding 95% confidence intervals (CI) to assess the association between comorbidities or other risk factors and outcomes.

Statistical significance was set at *p* < 0.05. SAS Enterprise Guide version 7.1 (SAS Institute Inc., Cary, NC, USA) was used for all statistical analyses.

## 3. Results

Baseline characteristics of patients undergoing AVF or AVG creation were analyzed according to SES (Table 1). Out of a total of 86,036 patients, 10,364 (12.1%) were in the MG, while 75,672 (87.9%) were in the HG. Within the HG, 75.4% received AVF and 24.6% received AVG, where as in the MG, 68.9% received AVF and 31.1% received AVG. Patients in the MG were significantly younger at the time of initial VA creation compared to those in the HG (60.4 ± 13.5 vs. 63.1 ± 13.6 years, respectively, *p* < 0.001).

Among patients aged 65 and older undergoing VA procedures, there were more individuals in the HG, with a higher proportion of men in this group (86.6% vs. 57.7%), while women were more prevalent in the MG (42.3% vs. 13.4%, *p* < 0.001). Comorbidities such as hypertension, diabetes, dyslipidemia, and peripheral arterial disease were significantly more prevalent in patients in the MG, whereas cancer was more prevalent in patients in the HG (all *p* < 0.05). The use of antiplatelets before VA creation was significantly higher in the MG (58.8% vs. 54.5%, respectively, *p* < 0.001). The MG had higher rates of visits to general hospitals and clinics compared to those in the HG (45.6% vs. 36.2% for general hospitals, 12.7% vs. 11.3% for clinics, respectively, *p* < 0.001).

There was no significant difference in mean body mass index (BMI) between the two groups, but patients with a BMI of 18.5 or higher were more prevalent in the HG. Blood pressure, creatinine level, and estimated glomerular filtration rate had a high proportion of missing values, but overall, patients in the HG were better managed. In terms of lifestyle habits, there were more smokers and drinkers in the MG.

Vascular access patency outcomes according to initial arteriovenous access type and health insurance status are presented in Table 2. Regarding AVF, primary, assisted primary, and secondary patency were all significantly worse in the MG (adjusted HR = 1.063, 95% CI: 1.027–1.100; adjusted HR = 1.149, 95% CI: 1.098–1.202; adjusted HR = 1.179, 95% CI; 1.093–1.274, respectively, all *p* < 0.001). For AVG, only assisted primary and secondary patency were significantly worse in the MG (adjusted HR = 1.092, 95% CI: 1.037–1.148, *p* < 0.001; adjusted HR = 1.164, 95% CI: 1.088–1.247, *p* < 0.001).

All-cause mortality according to initial arteriovenous access type and health insurance status is presented separately in Table 3. Regarding AVF, the mortality rate per 100 person-years was 9.24 for the HG and 11.66 for the MG. For AVG, the corresponding rates were 16.92 for the HG and 17.94 for the MG. Both crude and adjusted HRs were significantly higher in the MG for both AVF (crude HR = 1.259, 95% CI: 1.212–1.307; adjusted HR = 1.304, 95% CI: 1.253–1.355) and AVG (crude HR = 1.059, 95% CI: 1.007–1.114; adjusted HR = 1.212, 95% CI: 1.152–1.276), with all *p*-values being less than 0.05.

Table 4 summarizes the annual procedure counts and costs for VA revision, revealing significantly higher frequencies in the MG overall (2.10 vs. 1.87, *p* < 0.001). When specific procedures were examined, percutaneous thrombectomy was more frequently performed in the MG, resulting in significantly higher overall costs being incurred (0.26 ± 1.04 vs. 0.20 ± 0.83; USD 795.4 ± 3437.1 vs. USD 575.3 ± 2713.5, respectively, *p* < 0.001), while percutaneous angioplasty showed no significant difference between the HG and MG. Further stratification by VA type revealed a significant increase in procedures performed only in AVF (*p* = 0.003). Stent placement was also more frequent and costly in the MG (0.12 ± 0.45 vs. 0.10 ± 0.40; USD 549 ± 2684.9 vs. USD 462.5 ± 2499.6, respectively, *p* < 0.001). Although no significant difference was observed in vascular access type in AVF, the procedure costs were significantly higher for AVG in the MG (*p* = 0.039). Open surgical thrombectomy revision was more frequent and costly in the MG (0.47 ± 1.20 vs. 0.37 ± 1.03; USD 930.5 ± 3028.6 vs. USD 688 ± 2520.5, respectively, *p* < 0.001). These trends were consistent across both the AVF and AVG groups (all *p* < 0.001).

## 4. Discussion

In South Korea, the healthcare insurance system, operated by the NHIS, uniquely determines the costs of invasive procedures and establishes reimbursement policies through assessments by the Health Insurance Review and Assessment Service. This system categorizes patients into healthcare insurance beneficiaries and medical aid beneficiaries based on their SES, ensuring equitable access to care. While this system provides comprehensive coverage, patients under medical aid are generally considered economically disadvantaged.

ESKD patients on long-term hemodialysis are particularly vulnerable to financial and health challenges. The time-intensive nature of dialysis limits employment opportunities, contributing to a gradual economic decline [14]. In addition to socioeconomic burdens, VA dysfunction is a frequent complication during dialysis, requiring repeated and costly reinterventions. These procedures strain both healthcare resources and patient well-being, emphasizing the close interplay between SES and health outcomes.

Our study explored the relationship between SES and VA outcomes using a national cohort from South Korea’s NHIS. We found clear disparities in health status and healthcare utilization between patients covered by health insurance and those under medical aid. Notably, patients in the MG showed a higher prevalence of comorbidities, were younger at the time of initial VA creation, and demonstrated more frequent use of healthcare services. Conversely, cancer was more prevalent in the HG, suggesting a more complex interplay between SES and disease burden.

These differences in baseline characteristics are critical to understanding disparities in outcomes. The younger age at VA creation in the MG may reflect earlier onset of disease or lower access to preventive care. A higher comorbidity burden could exacerbate VA failure risk and increase the likelihood of repeated interventions. Meanwhile, the higher prevalence of cancer in the HG might indicate differences in screening access or age distribution. Together, these factors highlight how socioeconomic inequality shapes the clinical trajectory of hemodialysis patients and underscores the importance of tailored healthcare strategies.

VA types and outcomes also differed significantly by SES. The MG exhibited worse patency rates for both AVF and AVG, as well as significantly higher mortality rates [10,15]. Although comorbidities likely contributed to poorer outcomes, the disparities persisted even after adjustment, indicating that economic disadvantage independently impacts prognosis. Additionally, medical aid patients incurred higher VA-related healthcare costs.

Behavioral data from the NHIS revealed that over 75% of patients in the MG lacked results from national health screenings, which implies a substantial gap in engagement with preventive healthcare services. In contrast, only about half as many patients in the HG lacked such data, suggesting better access to or utilization of routine health checkups. This discrepancy may reflect barriers to preventive care among low-income populations, including limited health literacy, reduced access to primary care, or lower prioritization of health maintenance due to socioeconomic stressors. These gaps in screening can delay the identification and management of risk factors like smoking, hypertension, and diabetes, which are critical in preserving vascular access function. Therefore, strategies promoting lifestyle modification, early detection, and proactive risk management are especially vital for medically and economically vulnerable patients.

We also observed significant SES-based differences in VA reintervention rates. Patients in the MG underwent more frequent procedures for VA occlusion, such as surgical or percutaneous thrombectomy, compared to those in the HG, despite having similar rates of PTA. This suggests that MG patients may experience delayed clinical response to early signs of access dysfunction, possibly due to limited surveillance or inadequate patient awareness. The underutilization of pre-emptive angioplasty in the MG highlights systemic barriers to timely intervention, which may arise from resource constraints, lower continuity of care, or difficulties in navigating the healthcare system. Addressing these challenges through improved clinical monitoring and patient education is essential to reducing SES-related disparities in VA outcomes [16].

Given that medical aid recipients tend to seek care more frequently at general hospitals and clinics, strengthening clinical monitoring systems in these settings is critical. Implementing structured follow-up protocols, enhancing provider awareness, and promoting patient education could facilitate earlier detection and intervention for access dysfunction. These efforts are especially important, as the delayed recognition of vascular access failure often leads to more severe complications. Therefore, improving access to preventive care and ensuring timely clinical responses are essential to narrowing SES-based disparities in vascular access outcomes among hemodialysis patients.

Our study has some limitations, including its retrospective nature and reliance on administrative data, potentially introducing several biases: (1) selection bias arising from the retrospective data collection method; (2) misclassification bias due to inaccuracies in diagnostic coding; and (3) residual confounding bias from unmeasured variables such as detailed clinical parameters, medication adherence, and precise lifestyle modifications. Additionally, due to the conclusion of the data utilization period, we were unable to perform a sensitivity analysis excluding patients with missing baseline data (e.g., body mass index, blood pressure, creatinine, and substance use), which may affect the robustness of certain findings. Despite these limitations, our study underscores the critical role of socioeconomic status in shaping vascular access outcomes for hemodialysis patients in South Korea. Furthermore, our study benefits from utilizing a large national cohort, with comprehensive coverage by the NHIS database, reducing the risk of missing claims and patient loss.

## 5. Conclusions

Lower SES individuals experienced higher mortality rates and poorer VA patency, revealing significant disparities in VA outcomes among hemodialysis patients, despite equitable healthcare access and cost coverage in Korea’s healthcare system. These findings suggest that non-medical factors, including health literacy, adherence to treatment plans, and delays in seeking timely care, play a critical role in exacerbating these disparities. Addressing these issues requires targeted interventions aimed at improving preventive care, the early detection of VA complications, and patient education to enhance treatment adherence. Ultimately, comprehensive efforts to bridge these gaps are essential to ensure better outcomes and equity in vascular access care for vulnerable populations.

## Figures and Tables

**Table 1:** Baseline characteristics of patients with arteriovenous fistulas (AVFs) or arteriovenous grafts (AVGs) by socioeconomic status.

	Total *n* = 86,036	Health Insurance *n* = 75,672	Medical Aid *n* = 10,364	*p*-Value
VA type, *n* (%) AVF AVG	64,179 (74.6) 21,857 (25.4)	57,043 (75.4) 18,629 (24.6)	7136 (68.9) 3228 (31.1)	<0.001
Age of initial VA creation (years) 18–64 65–74 75–84 ≥85	62.8 ± 13.6 44,498 (51.7) 23,021 (26.8) 16,203 (18.8) 2314 (2.7)	63.1 ± 13.6 38,101 (50.4) 20,823 (27.5) 14,671 (19.4) 2077 (2.7)	60.4 ± 13.5 6397 (61.7) 2198 (21.2) 1532 (14.8) 237 (2.3)	<0.001 <0.001
Sex, *n* (%) Male Female	71,536 (83.1) 14,500 (16.9)	65,555 (86.6) 10,117 (13.4)	5981 (57.7) 4383 (42.3)	<0.001
Comorbidities, *n* (%) Hypertension Diabetes Cardiac disease Dyslipidemia Cerebrovascular disease Peripheral arterial disease Atrial fibrillation Cancer	76,622 (89.1) 56,175 (65.3) 2241 (2.6) 48,613 (56.5) 240 (0.3) 17,431 (20.3) 4058 (4.7) 8148 (9.5)	67,260 (88.9) 48,678 (64.3) 1983 (2.6) 42,597 (56.3) 218 (0.3) 15,000 (19.8) 3604 (4.8) 7373 (9.7)	9362 (90.3) 7497 (72.3) 258 (2.5) 6016 (58.0) 22 (0.2) 2431 (23.5) 454 (4.4) 775 (7.5)	<0.001 <0.001 0.432 0.001 0.170 <0.001 0.085 <0.001
Use of medication before creation, *n* (%) Direct oral anticoagulants Antiplatelet Anticoagulants	435 (0.5) 47,360 (55.0) 2615 (3.0)	391 (0.5) 41,271 (54.5) 2329 (3.1)	44 (0.4) 6089 (58.8) 286 (2.8)	0.215 <0.001 0.077
Hospital type, *n* (%) Tertiary hospital General hospital Clinic or medical center	44,071 (51.2) 32,116 (37.3) 9849 (11.4)	39,749 (52.5) 27,390 (36.2) 8533 (11.3)	4322 (41.7) 4726 (45.6) 1316 (12.7)	<0.001
Body mass index <18.5 ≥18.5 and <23 ≥23 and <25 ≥25 and <30 ≥30 NA	24.1 ± 3.6 1548 (1.8) 15,534 (18.1) 9905 (11.5) 12,983 (15.1) 2573 (3.0) 43,493 (50.6)	24.1 ± 3.6 1406 (1.9) 14,578 (19.3) 9410 (12.4) 12,270 (16.2) 2366 (3.1) 35,642 (47.1)	24.1 ± 4.2 142 (1.4) 956 (9.2) 495 (4.8) 713 (6.9) 207 (2.0) 7851 (75.8)	0.062 <0.001
Systolic blood pressure, mmHg <120 ≥120 and <140 ≥140 NA	137.2 ± 20.0 6491 (7.5) 17,859 (20.8) 18,146 (21.1) 43,540 (50.6)	137.4 ± 19.9 5977 (7.9) 16,866 (22.3) 17,180 (22.7) 35,649 (47.1)	134.5 ± 20.8 514 (5.0) 993 (9.6) 966 (9.3) 7891 (76.1)	<0.001 <0.001
Diastolic blood pressure, mmHg <80 ≥80 and <90 ≥90 NA	79.7 ± 12.3 18,678 (21.7) 14,222 (16.5) 9596 (11.2) 43,540 (50.6)	79.7 ± 12.3 17,558 (23.2) 13,407 (17.7) 9058 (12.0) 35,649 (47.1)	79.3 ± 12.4 1120 (10.8) 815 (7.9) 538 (5.2) 7891 (76.1)	0.099 <0.001
Creatinine, mg/dL ≤1.5 >1.5 NA	3.9 ± 3.4 7099 (8.3) 33,244 (38.6) 45,693 (53.1)	4.0 ± 3.5 6638 (8.8) 31,308 (41.8) 37,726 (49.9)	3.7 ± 2.9 461 (4.4) 1936 (18.7) 7967 (76.9)	<0.001 <0.001
eGFR, mL/min/1.73 m ≥60 <60 NA	25.6 ± 25.5 2795 (3.2) 29,500 (34.3) 53,741 (62.5)	25.4 ± 24.7 2578 (3.4) 27,535 (36.4) 45,559 (60.2)	28.4 ± 34.6 217 (2.1) 1965 (19.0) 8182 (78.9)	<0.001 0.013
Smoking Never smoker Previous smoker Current smoker No response NA	272 (0.3) 24,517 (28.5) 10,063 (11.7) 7691 (8.9) 43,493 (50.6)	238 (0.3) 23,203 (30.7) 9585 (12.7) 7004 (9.3) 35,642 (47.1)	34 (0.3) 1314 (12.7) 478 (4.6) 687 (6.6) 7851 (75.8)	<0.001
Alcohol use None Mild to moderate Heavy No response NA	311 (0.4) 32,609 (37.9) 8517 (9.9) 1106 (1.3) 43,493 (50.6)	274 (0.4) 30,569 (40.4) 8142 (10.8) 1045 (1.4) 35,642 (47.1)	37 (0.4) 2040 (19.7) 375 (3.6) 61 (0.6) 7851 (75.8)	<0.001

Categorical variables were analyzed using Pearson’s chi-square test. Continuous variables were analyzed using Student’s *t*-test. Values are presented as mean ± standard deviation or number (percentage). VA, vascular access; AVF, arteriovenous fistula; AVG, arteriovenous graft; NA, not available.

**Table 2:** Vascular access patency by initial arteriovenous access type and health insurance status.

	AVF (*n* = 64,179)			AVG (*n* = 21,857)		
	Health Insurance *n* = 57,043	Medical Aid *n* = 7136	*p*-Value	Health Insurance *n* = 18,629	Medical Aid *n* = 3228	*p*-Value
**Primary patency** No. of events Sum of person-years Incidence rate for 100 person-years (95% CI) Crude hazard ratio (95% CI) Adjusted hazard ratio (95% CI) *	29,887 125,609.7 23.79 (23.52–24.06) 1 (reference) 1 (reference)	3832 14,809 25.87 (25.06–26.71) 1.070 (1.034–1.106) 1.063 (1.027–1.100)	<0.001 <0.001	14,030 20,902.7 67.12 (66.01–68.24) 1 (reference) 1 (reference)	2410 3479.9 69.25 (66.52–72.08) 1.018 (0.975–1.063) 1.036 (0.992–1.082)	0.4178 0.1104
**Assisted primary patency** No. of events Sum of person-years Incidence rate for 100 person-years (95% CI) Crude hazard ratio (95% CI) Adjusted hazard ratio (95% CI) *	15,759 172,543.6 9.13 (8.99–9.28) 1 (reference) 1 (reference)	2198 20,130.6 10.92 (10.47–11.38) 1.170 (1.119–1.224) 1.149 (1.098–1.202)	<0.001 <0.001	9871 34,943.6 28.25 (27.69–28.81) 1 (reference) 1 (reference)	1780 5723.9 31.10 (29.67–32.58) 1.080 (1.024–1.135) 1.092 (1.037–1.148)	0.003 <0.001
**Secondary patency** No. of events Sum of person-years Incidence rate for 100 person-years (95% CI) Crude hazard ratio (95% CI) Adjusted hazard ratio (95% CI) *	5236 199,551.7 2.62 (2.55–2.70) 1 (reference) 1 (reference)	774 23,722.3 3.26 (3.04–3.50) 1.230 (1.142–1.328) 1.179 (1.093–1.274)	<0.001 <0.001	5188 45,064.811.51 (11.20–11.83) 1 (reference) 1 (reference)	1032 7394.9 13.96 (13.12–14.83) 1.198 (1.121–1.280) 1.164 (1.088–1.247)	<0.001 <0.001

Hazard ratios were estimated using Cox proportional hazards regression models. Adjusted hazard ratios were controlled for age, sex, hypertension, diabetes, cardiac disease, dyslipidemia, cerebrovascular disease, peripheral arterial disease, atrial fibrillation, cancer, type of creation hospital, BMI, systolic blood pressure, smoking, and alcohol consumption. * CI, confidence interval; AVF, arteriovenous fistula; AVG, arteriovenous graft.

**Table 3:** All-cause mortality by initial arteriovenous access type and health insurance status.

	AVF (*n* = 64,179)			AVG (*n* = 21,857)		
	Health Insurance *n* = 57,043	Medical Aid *n* = 7136	*p*-Value	Health Insurance *n* = 18,629	Medical Aid *n* = 3228	*p*-Value
**All-cause mortality** No. of events Sum of person-years Incidence rate for 100 person-years (95% CI) Crude hazard ratio (95% CI) Adjusted hazard ratio (95% CI) *	19,975 216,087.6 9.24 (9.12–9.37) 1 (reference) 1 (reference)	3054 26,194.8 11.66 (11.25–2.08) 1.259 (1.212–1.307) 1.304 (1.253–1.355)	<0.001 <0.001	9840 58,140.3 16.92 (16.59–17.26) 1 (reference) 1 (reference)	1797 10,015.4 17.94 (17.12–18.79) 1.059 (1.007–1.114) 1.212 (1.152–1.276)	0.0246 <0.001

Hazard ratios were estimated using Cox proportional hazards regression models. Adjusted hazard ratios were controlled for age, sex, hypertension, diabetes, cardiac disease, dyslipidemia, cerebrovascular disease, peripheral arterial disease, atrial fibrillation, cancer, type of creation hospital, BMI, systolic blood pressure, smoking, and alcohol consumption. * CI, confidence interval; AVF, arteriovenous fistula; AVG, arteriovenous graft.

**Table 4:** Comparison of vascular access revision procedures by health insurance status.

	Health Insurance			Medical Aid			*p*-Value (Health Insurance vs. Medical Aid)		
	Total (*n* = 75,672)	AVF (*n* = 57,043)	AVG (*n* = 18,629)	Total (*n* = 10,364)	AVF (*n* = 7136)	AVG (*n* = 3228)	Total	AVF	AVG
Percutaneous angioplasty No. Total cost, USD	1.20 ± 2.39 2376.1 ± 5430.9	0.95 ± 2.03 1776.1 ± 4465.6	1.97 ± 3.12 4212.4 ± 7367.6	1.24 ± 2.49 2518.1 ± 5676.4	0.94 ± 2.06 1735.9 ± 4234.7	1.90 ± 3.14 4247.1 ± 7712	0.314 0.286	0.332 0.220	0.112 0.209
Percutaneous thrombectomy No. Total cost, USD	0.20 ± 0.83 575.3 ± 2713.5	0.07 ± 0.42 211.6 ± 1542.1	0.59 ± 1.42 1690.3 ± 4580.7	0.26 ± 1.04 795.4 ± 3437.1	0.09 ± 0.55 244 ± 1462.5	0.65 ± 1.61 2013.1 ± 5572.2	<0.001 <0.001	0.003 0.003	0.354 0.225
Stent No. Total cost, USD	0.10 ± 0.40 462.5 ± 2499.6	0.07 ± 0.32 333.6 ± 2152.1	0.20 ± 0.57 858.7 ± 3315.1	0.12 ± 0.45 549 ± 2684.9	0.07 ± 0.34 330.5 ± 2164.5	0.22 ± 0.61 1031.7 ± 3529	<0.001 <0.001	0.090 0.109	0.066 0.039
Surgical thrombectomy with revision No. Total cost, USD	0.37 ± 1.03 688 ± 2520.5	0.20 ± 0.61 358.3 ± 1644.8	0.90 ± 1.68 1697.3 ± 4021.6	0.47 ± 1.20 930.5 ± 3028.6	0.24 ± 0.71 441.7 ± 1912	0.98 ± 1.76 2010 ± 4436.3	<0.001 <0.001	<0.001 <0.001	0.028 0.002
Any type of procedure No. (median [IQR]) Total cost, USD	1.87 (3.42) 4101.9 ± 9200	1.28 (2.56) 2679.5 ± 6572.2	3.66 (4.82) 8458.7 ± 13,652.5	2.10 (3.81) 4792.3 ± 10,449.4	1.35 (2.75) 2752.1 ± 6342.9	3.75 (5.08) 9301.9 ± 15,235.5	<0.001 <0.001	0.172 0.258	0.829 0.443

Continuous variables were analyzed using Student’s *t*-test. Values are presented as mean ± standard deviation. Cost values are in US dollars (USD 1 USD = KRW 1371). AVF, arteriovenous fistula; AVG, arteriovenous graft; IQR, interquartile range.

## Data Availability

The data that support the findings of this study are available from the National Health Insurance Service of Korea, but restrictions apply to the availability of these data, which were used under license for the current study, and so are not publicly available. Data are, however, available with permission of the National Health Insurance Service of Korea.

## Abbreviations

The following abbreviations are used in this manuscript:

SES	Socioeconomic status
VA	Vascular access
HG	Health insurance group
LD	Linear dichroism

## Appendix A

**Table A1:** CD-10 codes used to define covariates.

Covariates	ICD-10 Codes
Hypertension	I10
Diabetes	E10, E11
Coronary artery disease	I21, I22, M6599, M6593, M6594, M6601, M6602, M6630, M6636, M6637, O0226, M0227, O2066
Dyslipidemia	E78
Cerebrovascular disease	I63, I64, I65, I66, I678, O1641, OA641, O1640, OA640, O1648, OA648, O1649, OA649, O1647, OA647, O1642, OA642, M6551, M6553, M6554, M6552, M6561, M6563, M6564, M6565, M6566, M6567, M6562, M6564, M6571, M6572, M6634, M6638
Peripheral arterial disease	I70, I73
Atrial fibrillation	I48
Cancer	C-
